# Brain-targeting autoantibodies in patients with dementia

**DOI:** 10.3389/fneur.2024.1412813

**Published:** 2024-07-10

**Authors:** Finja Staabs, Helle Foverskov Rasmussen, Maria Buthut, Markus Höltje, Lucie Y. Li, Winfried Stöcker, Bianca Teegen, Harald Prüss

**Affiliations:** ^1^Department of Neurology and Experimental Neurology, Charité-Universitätsmedizin Berlin, Corporate Member of Freie Universität Berlin and Humboldt-Universität zu Berlin, Berlin, Germany; ^2^German Center for Neurodegenerative Diseases (DZNE) Berlin, Berlin, Germany; ^3^Institute of Integrative Neuroanatomy Berlin, Charité-Universitätsmedizin Berlin, Corporate Member of Freie Universiät Berlin and Humboldt-Universität zu Berlin, Berlin, Germany; ^4^Clinical Immunological Laboratory Prof. Stöcker, Groß Grönau, Germany

**Keywords:** neurodegenerative dementia, autoantibodies, CSF, cognitive impairment, autoimmunity

## Abstract

Autoantibodies against proteins in the brain are increasingly considered as a potential cause of cognitive decline, not only in subacute autoimmune encephalopathies but also in slowly progressing impairment of memory in patients with classical neurodegenerative dementias. In this retrospective cohort study of 161 well-characterized patients with different forms of dementia and 34 controls, we determined the prevalence of immunoglobulin (Ig) G and IgA autoantibodies to brain proteins using unbiased immunofluorescence staining of unfixed murine brain sections. Autoantibodies were detected in 21.1% of dementia patients and in 2.9% of gender-matched controls, with higher frequencies in vascular dementia (42%), Alzheimer’s disease (30%), dementia of unknown cause (25%), and subjective cognitive impairment (16.7%). Underlying antigens involved glial fibrillary acidic protein (GFAP), glycine receptor, and Rho GTPase activating protein 26 (ARHGAP26), but also a range of yet undetermined epitopes on neurons, myelinated fiber tracts, choroid plexus, glial cells, and blood vessels. Antibody-positive patients were younger than antibody-negative patients but did not differ in the extent of cognitive impairment, epidemiological and clinical factors, or comorbidities. Further research is needed to understand the potential contribution to disease progression and symptomatology, and to determine the antigenic targets of dementia-associated autoantibodies.

## 1 Introduction

Detection of anti-neuronal autoantibodies in clinical neurology has markedly changed routine assessment of patients with subacute neuropsychiatric abnormalities in the context of autoimmune encephalopathies. Lately, autoantibody diagnostics has been expanded to more chronic, slowly progressing changes of cognition, mood and behavior—where it allowed the recognition of treatment-responsive clinical entities previously thought to be classical neurodegenerative diseases (1).

For several of these autoantibodies, the direct pathogenicity has already been proven, such as for antibodies targeting the N-methyl-D-aspartate (NMDA)-receptor, ℽ-aminobutyric acid A (GABA_A_) and GABA_B_ receptors, α-amino-3-hydroxy-5-methyl-4-isoxazolepropionic acid (AMPA) receptor, Contactin-associated protein-like 2 (Caspr2) or leucine rich, glioma activated-1 (LGI1) (2). While many of them cause a broader clinical phenotype including epileptic seizures, psychosis or movement disorders, cognitive impairment and amnesia are common features and can predominate, which then overlaps with the clinical presentation of neurodegenerative dementias (3, 4). For example, patients with LGI1 autoantibodies can present to memory clinics with anterograde amnesia and behavioral abnormalities suggestive of Alzheimer’s disease (AD) or frontotemporal dementia (FTD) (5, 6). These patients often have post-inflammatory rapidly progressing atrophy in the mesiotemporal lobes (7), the predominant area of neuronal loss also in AD.

Another example is autoantibodies against the neuronal cell adhesion molecule Ig family containing LAMP, OBCAM, and NTM 5 (IgLON5), which causes a subacute encephalopathy with behavioral abnormalities and sleep disorder, but also cognitive impairment in up to 40% of affected patients (8, 9). Patients can have depositions of hyperphosphorylated *Tau* protein in hippocampus and brainstem, indicative of a primary neurodegenerative disease (tauopathy) (10). As clinical symptoms can respond to immunotherapy, the autoantibodies may be directly involved in the initiation or propagation of neurodegenerative processes, which is currently under intensive investigation.

The list of potential “dementia autoantibodies” is continuously growing and further contains autoantibodies against GFAP (11, 12), alpha1-adrenergic receptors (13), NMDA receptors (14–17), and multiple neuronal antigens in cancer patients with cognitive impairment (18). Initiation of immunotherapy in patients with LGI1 and IgLON5 autoantibodies can partially reverse the cognitive impairment, underscoring both, the need for and the potential of early autoantibody diagnostics in presumed neurodegenerative diseases. To further understand the role of such autoantibodies in dementia, detailed analyses of the frequencies, titers, kinetics, and pathogenicity of the antibodies are needed. Here, we systematically searched for established and novel anti-neuronal autoantibodies in cerebrospinal fluid (CSF) and serum of patients with different types of dementia using indirect immunofluorescence on unfixed murine brain sections.

## 2 Methods

### 2.1 Study population

**Figure 1:**
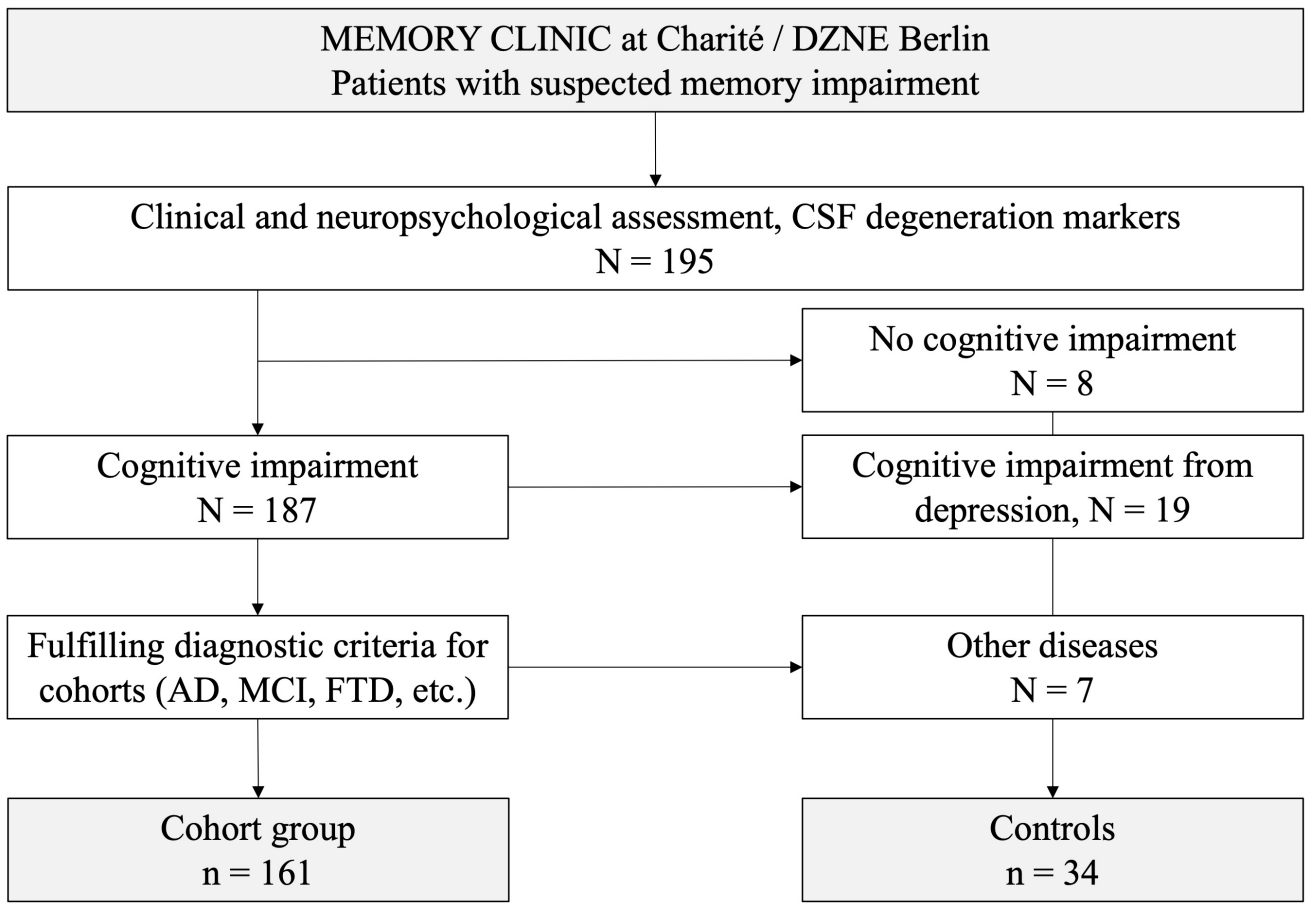
Recruitment of patients and controls into the study.

For anti-neuronal autoantibody testing, 195 patients with different forms of dementia and controls were recruited for this study from the memory clinic of the Department of Neurology at Charité Berlin, Campus Mitte (Figure 1). Patients were diagnosed according to current clinical guidelines (19) and assigned to one of the following diagnostic groups: (1) AD, (2) mild cognitive impairment due to AD (MCI), (3) FTD, (4) other [including Lewy body dementia (LBD), mixed dementia, and cerebral amyloid angiopathy], (5) vascular dementia, (6) cognitive impairment of unknown cause, and (7) subjective cognitive impairment (SCI). Patients with cognitive impairment due to depression or with neurological autoimmune disease, e.g., multiple sclerosis, were excluded. The control group consisted of 34 patients (Table 1) without neurodegenerative disease, including patients with depression and other psychiatric disorders (*n* = 19), patients with concerns of memory impairment but excluded neurodegenerative disease (*n* = 8), headache (*n* = 4), syncope (*n* = 1), minor stroke (*n* = 1), and paresthesia (*n* = 1).

**Table 1:** Clinical data, imaging and laboratory findings of dementia patients and controls.

	Dementia group			Control group	*p*-value	
	All	Antibody-positive	Antibody-negative	Controls	Ab-positive vs. -negative (dementia group)	All dementia vs. controls
Subjects (*n*)	161	34	127	34		
Age (mean + SD; median) (years)	72.6 ± 10.5; 76	68.3 ± 11.8; 73	73.8 ± 9.9; 76	60.7 ± 13; 61	***p* < 0.05**	***p* < 0.05**
Sex ratio (m:f)	88:73	18:16	70:57	19:15	*p* > 0.05	*p* > 0.05
MMSE (mean + SD; median) (points)	22 ± 6.3; 24	20.25 ± 7; 20.5	22.4 ± 6.1; 24	26.25 ± 3.7; 28	*p* > 0.05	***p* < 0.05**
Autoimmune disease (*n*)	16 (9.8%)	3 (11.5%)^a^	13 (9.5%)^c^	6 (25.7%)^e^	*p* > 0.05	*p* > 0.05
Cancer (*n*)	26 (16%)	1 (3.9%)^b^	25 (18.2%)^d^	4 (11.4%)^f^	*p* > 0.05	*p* > 0.05
**CSF**						
Phospho-Tau (mean) (<62 pg./mL)	78.7	84.3	77.1	45.2	*p* > 0.05	***p* < 0.05**
Total-Tau (mean) (<290 pg./mL)	572.8	622.1	558.6	246.9	*p* > 0.05	***p* < 0.05**
Beta-amyloid 1–40 (mean + SD)	15600.2	14857.7	15,813	11097.9	*p* > 0.05	***p* < 0.05**
Beta-amyloid 1–42 (mean + SD) (>629 pg./mL)	735.9	774.1	724.5	768.9	*p* > 0.05	*p* > 0.05
Amyloid ratio (mean + SD) (>0.069)	0.053	0.051	0.053	0.074	*p* > 0.05	*p* > 0.05
NfL (mean) (<1,300)	1824	1350.5	1981.9	703.8	*p* > 0.05	***p* < 0.05**
Cell count (mean) (0–4/μL)	2.2	2.7	2.2	5.9	*p* > 0.05	*p* > 0.05
Lactate (mean) (10–22 mg/dL)	15.2	15.8	14.9	14.9	*p* > 0.05	*p* > 0.05
Total protein count (mean) (150–450 mg/L)	501.4	442.6	523.1	514.9	*p* > 0.05	*p* > 0.05
Q-Albumin (mean)	7.6	6.8	7.9	7.8	*p* > 0.05	*p* > 0.05
CSF-specific oligoclonal bands	21 (20.2%)	5 (20%)	16 (20.3%)	2 (11.1%)	*p* > 0.05	*p* > 0.05
**Imaging**						
Imaging available	102	19	83	12		
No pathological findings	11	3	8	7	*p* > 0.05	***p* < 0.05**
Atrophy	68	12	56	3	***p* < 0.05**	***p* < 0.05**
Leukoencephalopathy	28	3	25	0	*p* > 0.05	***p* < 0.05**
Ischemia	17	3	14	0	*p* > 0.05	*p* > 0.05
Bleeding	10	3	7	0	*p* > 0.05	*p* > 0.05

^a^Lichen ruber planus (*n* = 1), Hashimoto’s thyreoiditis (*n* = 1), and myasthenia gravis (*n* = 1). ^b^Prostate cancer (*n* = 1). ^c^Hashimoto’s thyreoiditis (*n* = 3), CIDP (*n* = 2), vitiligo (*n* = 2), rheumatoid arthritis (*n* = 2), Crohn’s disease (*n* = 1), polymyalgia rheumatica (*n* = 1), psoriasis (*n* = 1), and myasthenia gravis (*n* = 1). ^d^Prostate cancer (*n* = 9), breast cancer (*n* = 3), colon cancer (*n* = 2), bladder cancer (*n* = 2), neuroendocrine tumor (*n* = 1), cutaneous B-cell lymphoma (*n* = 1), testicular cancer (*n* = 1), ovarian cancer (*n* = 1), chronic myelogenous leukemia (*n* = 1), rectal cancer (*n* = 1), thyroid cancer (*n* = 1), vestibular schwannoma (*n* = 1), basal cell carcinoma (*n* = 1), adenocarcinoma (*n* = 1), non-Hodgkin lymphoma (*n* = 1), astrocytoma (*n* = 1), and esophageal cancer (*n* = 1). ^e^Hashimoto’s thyroiditis (*n* = 2), vitiligo (*n* = 1), anti-phospholipid syndrome (*n* = 1), granulomatosis with polyangiitis (*n* = 1), rosacea (*n* = 1), and polychondritis trachea (*n* = 1). ^f^Breast cancer (*n* = 3), prostate cancer (*n* = 1). CSF, Cerebrospinal fluid; MMSE, Mini mental state examination; NfL, Neurofilament light chain; and SD, Standard deviation. Bold values represent the statistical significance.

Testing for an autoantibody panel using cell-based assays was available for the serum of 71 patients and the CSF of 36 patients. Serum from 189 and CSF from 34 patients was available for indirect immunofluorescence staining on murine unfixed brain sections. Serum and CSF were stored at −80°C until further use. Samples were pseudonymized and handled blinded to the status of the patients during the assessment and evaluation described below. The study was approved by the Charité Universitätsmedizin Berlin Institutional Review Board (Berlin, Germany, #EA1/258/18).

### 2.2 Tissue reactivity screening (indirect immunofluorescence)

To determine the prevalence of IgG and IgA isotype autoantibodies, serum and CSF were screened for tissue reactivity on 20 μm cryostat-cut unfixed sagittal mouse brain sections (C57BL/6 mice) as previously described (20–23). Briefly, sections were blocked for 1 h at room temperature in blocking solution [phosphate-buffered saline (PBS), pH 7.4, supplemented with 2% bovine serum albumin and 5% normal goat serum]. Serum (200 μL, diluted 1:400 in blocking solution) or CSF (200 μL, undiluted) were applied to the brain sections for 16 h at 4°C and washed with PBS. Bound antibodies were detected with goat anti-human IgG [Alexa Fluor®488 AffiniPure Goat Anti-Human IgG (H + L), Jackson ImmunoResearch, #109-545-003, dilution 1:1,000] or goat anti-human IgA (Fluorescein AffiniPure Goat Anti-Human IgA, Jackson ImmunoResearch #109-095-011, dilution 1:200). After 2 h of incubation at room temperature, sections were rinsed again and mounted with Immumount (Shandon, #9990402). Images were taken with fluorescence microscopes (Olympus CKX41, Leica DMI8/SPE, Nikon Scanning Confocal A1Rsi+).

Cerebrospinal fluid samples were additionally screened for established autoantibodies using commercial panel tests (Euroimmun AG, Lübeck, Germany), including Hu, Ri, anti-neuronal nuclear antibodies 3 (ANNA3), Yo, Anti-Tr/anti-Delta/Notch-like epidermal growth factor-related receptor (Tr/DNER), myelin, Ma/Ta, glutamate decarboxylase 65 (GAD65), amphiphysin, glutamate receptor type AMPA, GABA_B_ receptor, LGI1, Caspr2, zinc finger protein 4 (ZIC4), dipeptidyl aminopeptidase-like protein (DPPX), carbonic anhydrase related protein VIII (CARPVIII), glycine receptor (GlyR), metabotropic glutamate receptor 1 and 5 (mGluR1, mGluR5), GABA_A_ receptor, ARHGAP26, inositol 1,4,5-trisphosphate receptor type 1 (ITPR1), homer3, potassium voltage-gated channel subfamily A member 2 (KCNA2), myelin oligodendrocyte glycoprotein (MOG), recoverin, neurochondrin, glutamate receptor δ2 (GluRD2), flotillin-1/2, and IgLON5.

### 2.3 Evaluation criteria

Indirect immunofluorescence sections were evaluated according to a modified semi-quantitative fluorescence score ranging from 0 to 3, as previously described (24). “0” defined the absence of any fluorescence signal, “1” the faint “background” intensity commonly seen with serum of healthy controls, “2” a clearly visible fluorescence patterns with reproducible anatomical distribution, and “3” a high-intensity fluorescent staining as in positive controls. Consistent intensities ≥2 in repeated experiments were considered positive staining.

The frequently observed staining of neuronal nuclei often corresponded to established anti-nuclear antibodies (ANA, e.g., finely speckled nuclear ANA) (25) and was considered positive only if it was detected with an intensity of ≥2 in CSF. Co-staining with an anti-GFAP antibody (NeuroMab, Cat# 75–240, RRID:AB 10672299; dilution 1:1,000) was performed when a glia-like pattern was observed.

### 2.4 Statistical analysis and figures

**Figure 2:**
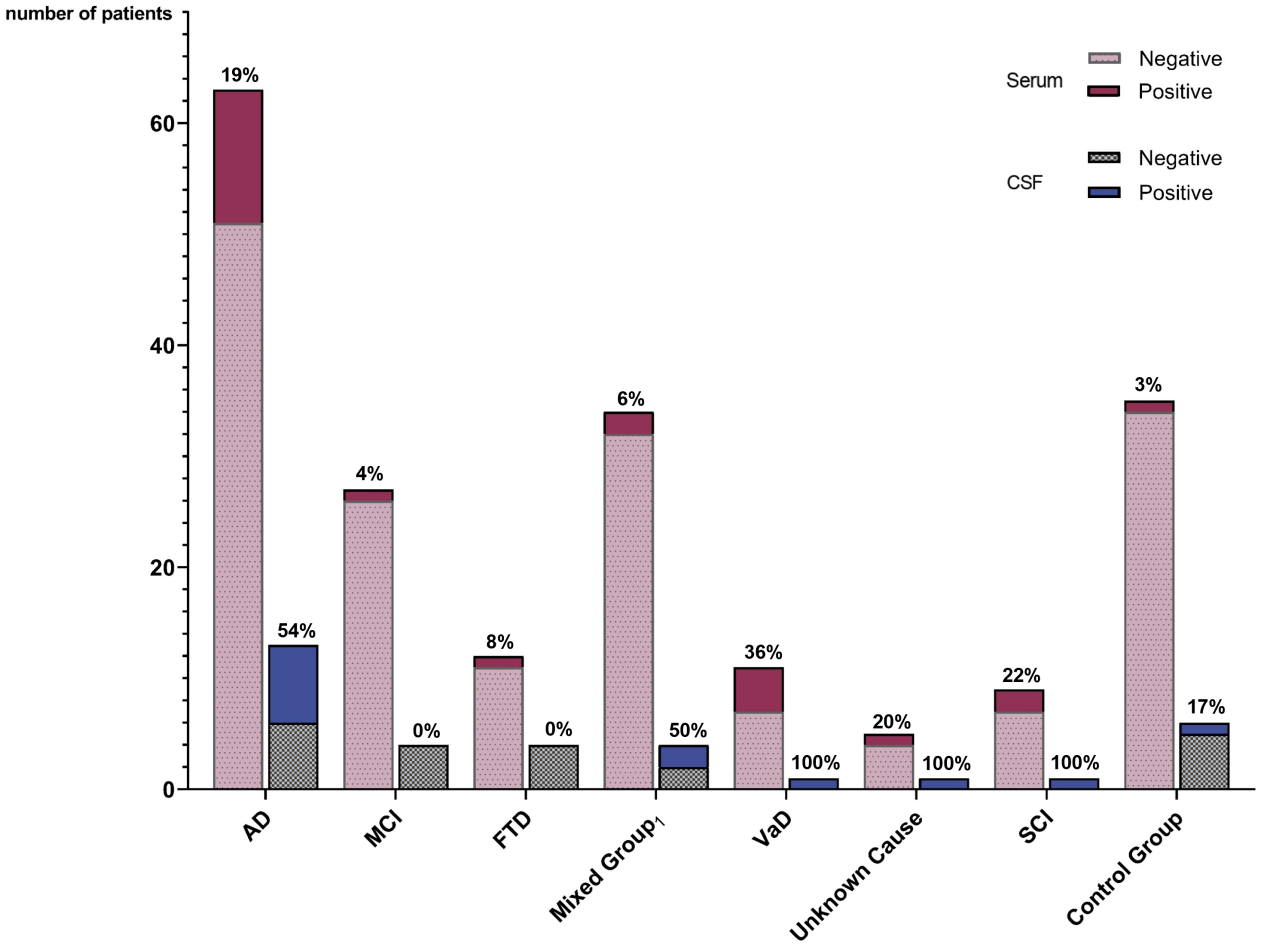
Prevalence of autoantibody-positive patients among different diagnostic groups. The percentage of positive patients within the diagnostic group is given for serum and for CSF, respectively. Lewy body dementia, mixed dementia, and amyloid angiopathy. AD, Alzheimer’s disease; MCI, Mild cognitive impairment; FTD, Frontotemporal dementia; VaD, Vascular dementia; SCI, Subjective cognitive impairment; CG, Control group; and CSF, Cerebrospinal fluid.

Statistics were conducted in Microsoft Excel (RRID: SCR_016137) including XLSTAT (RRID: SCR_016299). Statistical significance was assumed when *p* < 0.05. Differences in the prevalence of antibody-positive patients in the dementia cohort compared to controls were calculated using the Chi square test. Clinical parameters, including CSF markers, age and mini-mental state examination (MMSE) were compared using *t*-test, assuming unequal variances (Welch’s test). ANOVA was used for subgroup analysis. Figure 2 was created in GraphPad Prism (RRID: SCR_002798).

## 3 Results

### 3.1 Clinical data

Epidemiological and clinical data, CSF and imaging findings of dementia patients and gender-matched controls are shown in Table 1. The control cohort was younger than the dementia group (mean age 60.9 vs. 72.5 years). Basic CSF diagnostics (e.g., cell count, protein) were available for 89 dementia patients (55.3%) and 10 controls (29.4%). MMSE test results were available for 107 dementia patients (66.5%) and eight controls (23.5%) and showed lower values in the dementia group, as expected. Autoimmune diseases were more frequent in the control cohort (17.6 vs. 9.3%), while cancer was more prevalent in the dementia cohort (16.1 vs. 11.8%), not reaching statistical significance.

### 3.2 Prevalence of autoantibodies

IgG isotype autoantibodies were significantly more frequent in dementia patients (34 of 161 = 21.1%) compared to controls (1 of 34 = 2.9%, *p* = 0.04) (Table 2; Figure 2). Antibody-positive patients were younger compared to antibody-negative patients (*p* = 0.01; Table 1), while they did not differ significantly in MMSE scores, epidemiological and clinical factors, CSF and imaging parameters, or comorbidities (Table 1).

**Table 2:** Staining results and clinical data distributed among different diagnostic groups.

	AD		MCI		FTD		Other^a^		VaD		Unknown cause		SCI		Controls	
Subjects (*n*)	**63**		**28**		**12**		**35**		**12**		**5**		**6**		**35**	
Serum/CSF	63	12	28	4	12	4	32	5	11	1	5	1	6	1	32	6
Antibody-positive in serum/CSF (IIFT plus CBA)	13	6	2	0	3	0	2	3	4	1	0	1	0	1	1	1
Antibody-positive (%)	**17 (27%)**		**2 (7.1%)**		**3 (25%)**		**5 (14.3%)**		**5 (41.7%)**		**1 (25%)**		**1 (16.7%)**		**1 (2.9%)**	
Age (mean + SD) (years)	73.1 ± 9.1		74.1 ± 11		60.8 ± 9		75.2 ± 9.6		74.7 ± 11.9		65.2 ± 14		71 ± 10.6		60.7 ± 13	
MMSE (mean + SD) (points)	19 ± 7.2		26 ± 2.3		21 ± 5.9		22.2 ± 5		23.75 ± 5.1		19.3 ± 5.9		29.5 ± 0.5		26.33 ± 3.7	
Sex ratio (m:f)	31:32		16:12		6:6		20:15		10:2		3:2		2:4		19:16	
Cancer	12 (19.1%)		5 (17.9%)		2 (16.7%)		4 (11.4%)		2 (16.7%)		1 (25%)		0		4 (11.4%)	
Autoimmune disease	7 (11.11%)		1 (3.6%)		1 (8.3%)		3 (8.6%)		1 (8.3%)		1 (25%)		1 (16.7%)		6 (17.1%)	
**Imaging**																
Imaging available	38 (60.3%)		16 (57.1%)		10 (83.3%)		23 (65.7%)		10 (83.3%)		2 (40%)		3 (50%)		13 (37.1%)	
No pathological findings	5 (7.9%)		2 (12.5%)		1 (8.3%)		3 (8.6%)		0		0		0		8 (22.9%)	
Atrophy	29 (46%)		8 (50%)		9 (75%)		13 (37.1%)		4 (33.3%)		2 (40%)		3 (50%)		3 (8.6%)	
Leukoencephalopathy	7 (11.1%)		4 (25%)		0		11 (31.4%)		5 (41.7%)		0		2 (33.3%)		0	
Ischemia	6 (9.5%)		2 (12.5%)		0		7 (20%)		2 (16.7%)		0		0		0	
Bleeding	1 (1.6%)		1 (6.3%)		0		5 (14.3%)		3 (25%)		0		0		0	
Other	1 (1.6%)		2 (12.5%)		0		2 (5.7%)		2 (16.7%)		0		0		1 (2.9%)	
**CSF**																
pTau (mean + SD) (<62 pg./mL)	103.8 ± 37.5		67.3 ± 21.8		66.8 ± 49.5		59.7 ± 30.8		58.4 ± 17.8		71.7 ± 40.4		57.2 ± 35.5		45.2 ± 25.7	
tTau (mean + SD) (<290 pg./mL)	783.3 ± 406.8		445.1 ± 182.2		531.7 ± 550.3		393.7 ± 272.9		410.5 ± 259.2		479.4 ± 358.9		351.4 ± 251.3		246.9 ± 179.4	
Beta-Amyloid 1–40 (mean + SD)	17073.1 ± 7432.3		15632.3 ± 6624.1		11905.5 ± 6691.6		15640.7 ± 11094.6		13383.9 ± 3,650		11399.3 ± 1930.1		18,232 ± 8540.6		11097.9 ± 3762.4	
Beta-Amyloid 1–42 (mean + SD) (>629 pg./mL)	589 ± 205.7		993 ± 452.6		875.9 ± 350		715.8 ± 300.3		953.3 ± 468.5		756 ± 220.6		833.3 ± 201.6		768.9 ± 282.3	
Amyloid ratio (mean + SD) (>0.069)	0.036 ± 0.01		0.062 ± 0.034		0.09 ± 0.04		0.06 ± 0.03		0.067 ± 0.035		0.068 ± 0.028		0.028 ± 0.02		0.074 ± 0.031	
NfL (mean + SD) (<1,300)	1810.4 ± 727		2,153 ± 1438.5		3,195 ± 912.6		527.4 ± 488.5		-		-		-		703.8 ± 469.4	
Cell count (mean + SD) (0–4/μL)	1.7 ± 1.3		2.8 ± 2.3		1.8 ± 1.3		2.5 ± 5		3.25 ± 2.3		1.5 ± 1.2		2.6 ± 2.1		5.9 ± 14.6	
Lactate (mean + SD) (10–22 mg/dL)	14.9 ± 4.8		14. 9 ± 4.2		15.7 ± 2		15.8 ± 6.3		13.9 ± 5		15.3 ± 3.3		16.7 ± 3.7		13.4 ± 6	
Total protein count (mean + SD) (150–450 mg/L)	454.2 ± 187.5		452.2 ± 103.2		438.8 ± 167.4		598.1 ± 403.2		540 ± 210		675.5 ± 215.8		357.7 ± 85.2		514.9 ± 353.4	
Q-Albumin (mean + SD)	7 ± 3.4		6.8 ± 2.5		6.7 ± 2.5		8.4 ± 4.7		9.8 ± 4.4		9.9 ± 3		5.1 ± 1.3		7.8 ± 5.9	
CSF-specific OCB	27%		33%		11%		11%		11%		20%		25%		11%	

^a^Lewy body dementia, mixed dementia, and amyloid angiopathy. AD, Alzheimer’s disease; MCI, Mild cognitive impairment; FTD, Frontotemporal dementia; VaD, Vascular dementia; SCI, Subjective cognitive impairment. NfL, Neurofilament light chain; SD, Standard deviation; IIFT, Indirect immunofluorescence test; CBA, Cell-based assay; and CSF, cerebrospinal fluid. Bold values represent the sample size for better readability.

Statistical comparison of the individual dementia groups showed differences in autoantibody frequencies and clinical characteristics such as age, sex, and comorbidities (Table 2). Age and MMSE varied between groups (ANOVA, *p* < 0.001). The highest autoantibody prevalence was observed in patients with vascular dementia (41.7%) but was also high in AD (30%) and dementia of unknown cause (25%), while lowest in MCI (2.1%).

**Figure 3:**
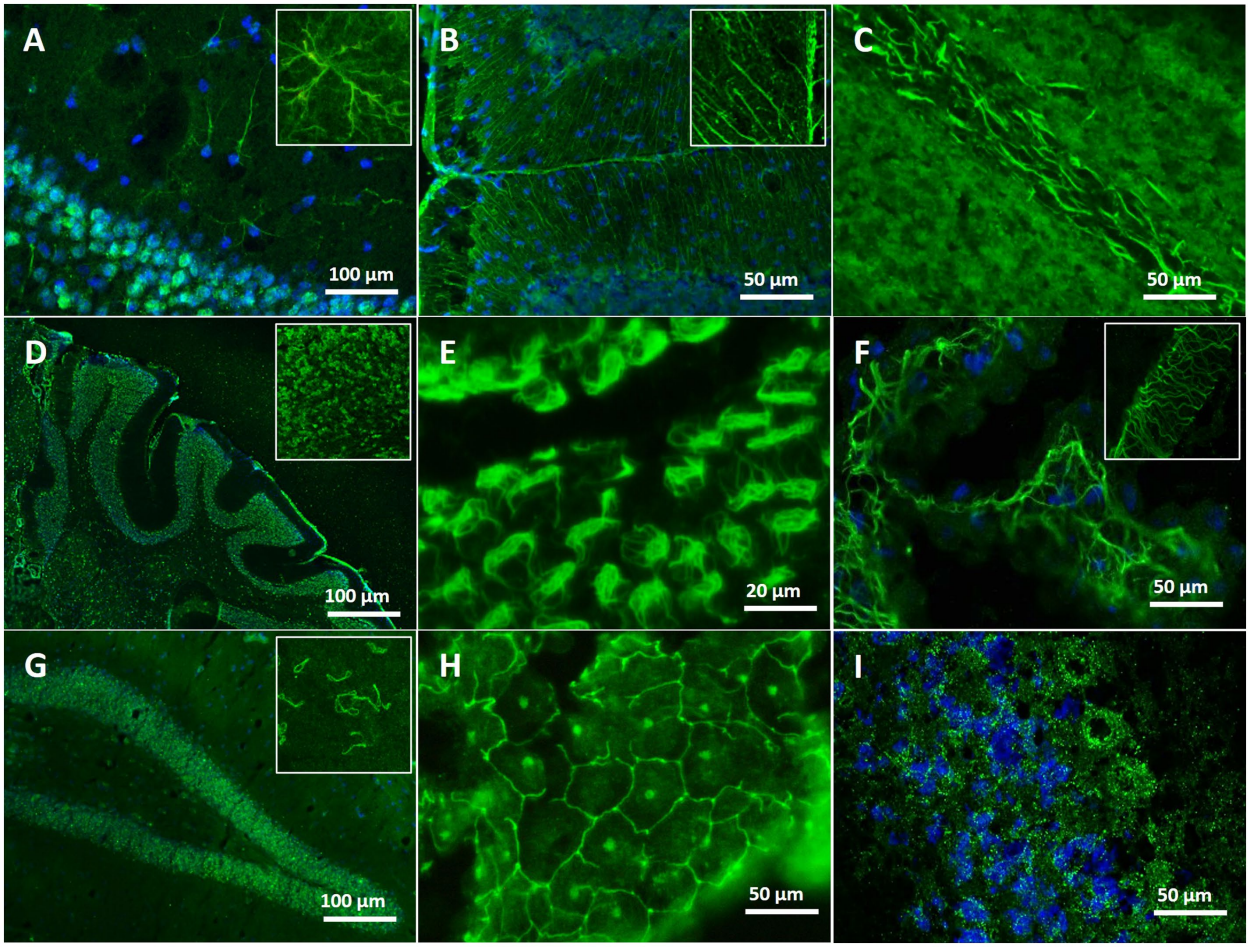
Immunofluorescence staining patterns on unfixed mouse brain with serum/CSF of different patients. **(A)** Staining of astrocytes in the hippocampal region (blue: co-staining of the nuclei with DAPI) with serum of an AD patient. **(B)** Distinct labeling of Bergmann glia cells in the cerebellum with serum of an AD patient. **(C)** Intense myelin staining in the deep white matter of the cerebellum with CSF of an AD patient. **(D)** Survey micrograph showing ubiquitous ANA staining in the cerebellum using serum of an AD patient. **(E)** Distinct staining of the ependymal layering of brain ventricles with serum of an AD patient. **(F)** Net-like staining in the choroid plexus with serum of an AD patient. **(G)** Intense ANA staining of rings and rod-like structures in hippocampus and cortex using CSF of an AD patient. **(H)** Reticular labeling of choroid plexus cells resembling a tight junction pattern, in addition staining of nucleoli, using serum of an MCI patient. **(I)** Finely speckled, granular ANA staining in the cerebellum with serum of an SCI patient (blue: co-staining of the nuclei with DAPI).

The patterns of autoantibody binding on unfixed murine brain sections in the 34 dementia patients ranged from myelin staining in cerebellum and/or thalamus (*n* = 11, Figure 3C) to astrocytes (*n* = 2, Figures 3A,B), brain blood vessels and immunostaining of the choroid plexus (*n* = 5, Figures 3E,F,H). Several of these patients had additional ANA patterns in the CSF (*n* = 10) and serum (*n* = 17) (Figures 3G,I), but also various staining patterns of fibers and structures with unknown target antigen (Table 3). ANA patterns included among others “rings and rods” and fine-speckled patterns. The single autoantibody-positive control patient showed binding to myelin and nerve fibers. Two further controls had ANA in serum, but not in CSF, thus being negative according to our criteria (Figure 3D).

**Table 3:** Distribution of staining patterns (intensity ≥2) on unfixed murine brain sections among the autoantibody-positive patients of all diagnostic groups.

		AD	MCI	FTD	Other^a^	VaD	Unknown cause	SCI	Controls
Positive patients (*n*)		**12**	**1**	**1**	**4**	**5**	**1**	**1**	**1**
	Net-like choroid plexus pattern	3	0	0	1	1	0	0	0
	Myelin	5	1	1	1	2	0	0	1
	Astrocytes	0	0	0	1	1	0	0	0
	Fiber-like staining	3	0	1	0	1	0	0	0
	Vessel staining	2	1	0	0	1	0	0	0
	Purkinje cells	1	1	0	0	0	0	0	0
	Other pattern	1^1^	1^2^	0	0	0	0	0	0
	ANA								
	Nucleoli	6	0	0	2	1	1	0	1
	Rings and rods	5	0	0	1	0	0	0	0
	Other ANA	5	0	0	1	0	0	1	0

^a^Lewy body dementia, mixed dementia, and amyloid angiopathy. ^1^Bergmann glia cells in cerebellum; ^2^Binding to vessels and choroid plexus, unknown target. AD, Alzheimer’s disease; MCI, Mild cognitive impairment; FTD, Frontotemporal dementia; VaD, Vascular dementia; SCI, Subjective cognitive impairment; and ANA, Anti-nuclear antibodies. Bold values represent the sample size for better readability.

The search for IgA isotype autoantibodies revealed only one positive patient (0.6%) with ANA reactivity in CSF. This patient was also positive for IgG autoantibodies.

Panel diagnostics for established autoantibodies using a commercial cell-based assay were available in 77 patients (30 CSF plus serum, 41 serum only, and six CSF only). Eleven (14.1%) were autoantibody-positive in serum, and included autoantibodies against myelin (*n* = 3; titers 1:100 to 1:320), GlyR (*n* = 2; 1:32), GABA_B_R (*n* = 2; 1:10 to 1:100), ARHGAP26 (*n* = 1; 1:32), GFAP (*n* = 1; 1:100), KCNA2 (*n* = 1; 1:320), and Caspr2 (*n* = 1; 1:32).

## 4 Discussion

In this pilot study, we identified both, well-established and potentially novel autoantibodies in 21.1% of patients with different forms of cognitive impairment and dementia, compared to 2.9% of controls. Antigenic targets included GFAP, ARHGAP26, KCNA2, Caspr2, GlyR, and GABA_B_R, but also various not yet identified antigens on unfixed mouse brain sections, such as myelinated fibers, brain vessels, astroglia, choroid plexus, and antinuclear antigens. Autoantibody prevalence was highest in vascular dementia (41.7%), but also common in AD (30%). Given the large variety of autoantibodies, it is not surprising that autoantibody-positive patients had similar MMSE scores compared to autoantibody-negative patients. Further studies are needed to identify potential relationships between certain subgroups of autoantibodies with clinical symptoms including cognitive impairment.

The here observed frequencies are in a similar range to previous studies. For example, in a recent cohort of 349 patients with various neurodegenerative diseases, established autoantibodies overlapping with our diagnostic panel were detected in 11.8% (26). Likewise, 13.8% of 93 patients with neurodegenerative disorders in another study had surface-reactive autoantibodies, although less common in AD (27). Focusing on NMDAR autoantibodies, an early study from our center reported 16.1% seropositivity in 286 patients with neurodegenerative dementia, with the highest prevalence in the subgroup of unclassified dementia (15).

Detection of autoantibodies in patients with cognitive impairment is clearly not sufficient for the classification of dementia as being “autoimmune,” as even well-characterized autoantibodies also regularly occur in control populations, at least in serum (26). The concept of autoimmune dementia so far embraces conditions with predominant memory impairment typically characterized by subacute onset, a rapid, fluctuating progression and inflammatory CSF parameters, often with detected anti-neuronal autoantibodies and responsiveness to immunotherapy (3, 28, 29). In some patients with autoimmune dementia, neurodegeneration biomarker profiles can mimic protein patterns indicative of neuronal destruction seen in neurodegenerative dementias. On the other hands, autoantibodies may not always cause damage, but could be mere bystanders or even convey positive effects, ranging from limiting damage to reducing neurodegeneration-associated proteins such as β-amyloid, or facilitating remyelination (30).

It is subject of intensive research, which autoantibodies may contribute to cognitive impairment and how they exert effects. The current study did not assess pathogenic functions of the identified autoantibodies. For some of them, however, previous studies demonstrated pathogenicity that can be plausibly linked to cognitive impairment, even though numbers of study participants were generally low. For example, cognitive impairment was the main common feature of five patients with GlyR autoantibodies in one study, associated with elevated tTau/pTau in CSF (31). Similarly, in our analysis, the two patients with serum GlyR autoantibodies had the diagnosis of early-stage AD with increased pTau/tTau levels. Autoantibodies against KCNA2 have also been reported in progressive dementia, in one case with an AD CSF profile (32). In patients with FTD, autoantibodies against GluR3 and IgLON5 were found (33, 34), which are known to induce receptor internalization and impair long-term synaptic plasticity (35, 36). We could further identify several dementia patients with autoantibodies against GFAP and/or astrocytes, which seems an interesting new marker not only for a subacute autoimmune meningoencephalomyelitis (37–40), but also for patients with slowly progressing cognitive decline and dementia (41–44).

Autoantibodies in our study were not equally distributed between subgroups of patients. In AD patients, 30% had IgG binding to certain brain antigens, which is in line with previous findings of a wide range of autoantibodies observed in AD, such as against NMDAR, dopamine receptor, acetylcholine receptor, and many more using targeted and unbiased detection approaches (12, 14, 15, 45–50). The highest prevalence was observed in vascular dementia with several patients harboring not previously described autoantibodies against the choroid plexus. Whether such autoantibodies can impair blood–brain barrier function and potentially dysregulate permeability for neurotoxic molecules is currently unclear. However, studies on autoantibodies targeting endothelial barrier function in the brain suggest that this can be a pathogenic mechanism (51–54).

The frequent finding of ANAs adds to previous studies which, for example, found significantly increased serum frequencies in patients with FTD (60% versus 13% in healthy controls) (55). Data on ANAs in CSF are almost not available. We thus focused on CSF and found 20.5% of available CSF samples from the dementia cohort to be positive for ANAs. Although ANAs have long been considered non-pathogenic due to their intracellular targets, increasing evidence suggests that autoantibodies can reach intracellular epitopes and can have disease-related effects (56, 57). Development of pathology may take much longer compared to binding of autoantibodies to neuronal surface receptors. It is tempting to speculate, however, whether such protracted subtle effects may build up over time and contribute to cognitive impairment when patients at risk have high-level autoantibodies, a concept that was recently coined “smoldering humoral autoimmunity” (1).

The study has several limitations. Related to the retrospective study design, CSF and serum samples, clinical information, neuropsychological assessments and imaging were not available for the entire cohort. Despite the number of 195 study participants, some disease subgroups were too small for a robust statistical analysis, in particular as the patients showed variability in disease course and comorbidities. The age difference between dementia patients and controls is likely related to the preferential age of the different diseases, however, it may have affected our findings as (humoral) autoimmunity and inflammation is age-dependent. Although our diagnostic assay using unfixed murine brain sections has been consistently used in different neuropsychiatric clinical conditions to identify novel autoantibodies [e.g. (21, 22, 58, 59)], the handling of unfixed brain has technical challenges, which so far prevented broader application in routine laboratories, thus validation across different centers is pending.

Taken together, the present study reports increased frequencies of established and novel autoantibodies in patients with cognitive impairment, suggesting that many more autoantibodies can be seen in the CSF of patients with cognitive impairment, than currently investigated using routine assays. Evolving data on immunotherapy-responsive cases suggest that some antibodies are not mere bystanders of dysregulated autoimmunity following neurodegeneration. Better understanding of the autoantibodies’ function will help to identify future patients who might benefit from treatment. The generation of human disease-derived monoclonal autoantibodies will likely change our approach to autoimmune dementia in the near future, as we will learn about the pathogenicity of these antibodies, their antigenic targets on neurons and glial cells, their contribution to disease, and how they can facilitate the development of novel, antibody-selective therapies.

## Data availability statement

The original contributions presented in the study are included in the article/supplementary material, further inquiries can be directed to the corresponding author.

## Ethics statement

The studies involving humans were approved by Charité Universitätsmedizin Berlin Institutional Review Board, #EA1/258/18. The studies were conducted in accordance with the local legislation and institutional requirements. The participants provided their written informed consent to participate in this study.

## Author contributions

FS: Conceptualization, Data curation, Formal analysis, Investigation, Methodology, Writing – original draft, Writing – review & editing. HF: Data curation, Writing – original draft, Writing – review & editing. MB: Data curation, Supervision, Writing – review & editing. MH: Supervision, Writing – review & editing. LL: Data curation, Writing – review & editing. WS: Data curation, Investigation, Writing – review & editing. BT: Data curation, Investigation, Writing – review & editing. HP: Conceptualization, Data curation, Funding acquisition, Investigation, Methodology, Project administration, Resources, Supervision, Writing – original draft, Writing – review & editing.
